# Oblique rift opening revealed by reoccurring magma injection in central Iceland

**DOI:** 10.1038/ncomms12352

**Published:** 2016-08-05

**Authors:** Joël Ruch, Teng Wang, Wenbin Xu, Martin Hensch, Sigurjón Jónsson

**Affiliations:** 1 King Abdullah University of Science and Technology (KAUST), Physical Science and Engineering Division (PSE), Thuwal 23955-6900, Saudi Arabia; 2 Icelandic Meteorological Office, IS-150 Reykjavik, Iceland

## Abstract

Extension deficit builds up over centuries at divergent plate boundaries and is recurrently removed during rifting events, accompanied by magma intrusions and transient metre-scale deformation. However, information on transient near-field deformation has rarely been captured, hindering progress in understanding rifting mechanisms and evolution. Here we show new evidence of oblique rift opening during a rifting event influenced by pre-existing fractures and two centuries of extension deficit accumulation. This event originated from the Bárðarbunga caldera and led to the largest basaltic eruption in Iceland in >200 years. The results show that the opening was initially accompanied by left-lateral shear that ceased with increasing opening. Our results imply that pre-existing fractures play a key role in controlling oblique rift opening at divergent plate boundaries.

## Abstract (web summary)

Deformation at divergent plate boundaries during rifting events is rarely observed and therefore poorly constrained. Ruch *et al*. show evidence of an oblique rift opening at the Bárðarbunga caldera, Iceland, which was one of the largest basaltic eruptions observed in the last 200 years

## Graphical abstract

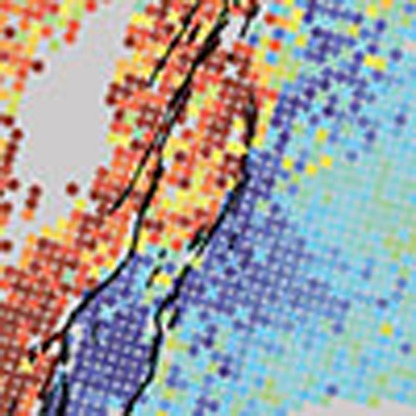

The active rift in Iceland is the locus where the Eurasian plate separates from the North American plate at a rate of 18.5 mm per year in the N104°E direction^1^. Two transform fault zones, the South Iceland Seismic Zone to the south and the Tjörnes Fracture Zone to the north, bound most of the rift in Iceland, often referred to as the Eastern and Northern Volcanic Zones^2^. The plate boundary in Iceland consists of roughly 30 spreading centres, of which 12 are characterized by a central volcano and an associated fissure swarm^2^. The fissure swarms are used as a pathway for magma that propagates laterally from the plumbing system of the central volcano during rifting events, generating large deformation, normal faulting and sometimes eruptions^3^,^4^,^5^.

The Bárðarbunga spreading centre is located at the transition between the Eastern and the Northern Volcanic Zones (Fig. 1a) and is known for producing very large effusive lava eruptions^2^,^6^. The northern section of the Bárðarbunga fissure swarm is not well-defined as it is partly covered by the Vatnajökull ice cap and its alluvial flood plains. Some volcanic features (for example, cone rows, lava flows and a partly buried fissure swarm) have, however, been mapped and the recent 2014–15 eruption remarkably used the same eruptive vents as the earlier 1797 eruption^7^,^8^.

Here we study the near-field deformation and faulting activity near the 2014–15 eruption site using radar-amplitude-image offsets, earthquake fault plane solutions and field structural data. The results show that the rifting event reactivated a pre-existing graben and that the opening was accompanied with significant left-lateral shear. Together, the oblique rifting opening, the graben reactivation and the reuse of eruptive vents illustrate the importance of pre-existing fractures in controlling magma migration at divergent plate boundaries.

## Results

### Timing and seismicity of the 2014–15 intrusion

**Figure 1:**
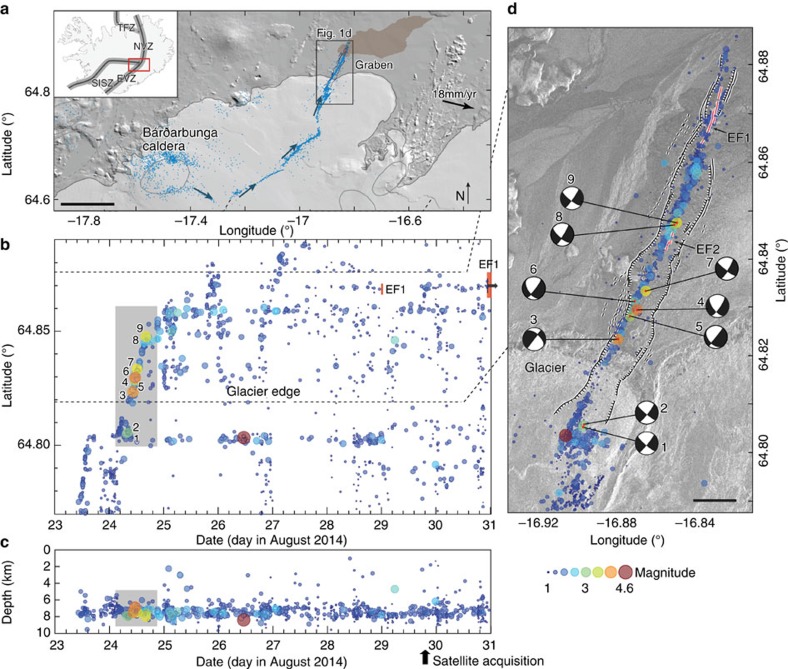
Rifting event seismicity and graben location. (**a**) Map of the study area in central Iceland (see inset) showing the seismic swarm pathway (blue dots and arrows) from Bárðarbunga caldera to the eruption site north of the glacier. The brown shaded area shows the lava flow coverage as in October 2014. EVZ, East Volcanic Zone; NVZ, North Volcanic Zone; SISZ, South Iceland Seismic Zone; TFZ, Tjörnes Fracture Zone. Black scale bar, 10 km. (**b**) Earthquake latitude and (**c**) earthquake depths as a function of time referenced to sea level for the northernmost part of the earthquake sequence^7^. The colour and size of each circle is according to earthquake magnitude. The red vertical bars mark the timing of the principal eruptive fissure activity EF1. The grey rectangles mark the period of the most intensive earthquake activity with numbered events further described in **d** and Supplementary Fig. 1. The first satellite image acquisition was on 29 August at 19:37:41 GMT. (**d**) SAR amplitude-image map of the graben showing earthquake epicentres, fault plane solutions for *M*_w_>3.5 events and eruptive fissures (EF1 and EF2). Black scale bar, 1 km.

We first analysed the temporal development of the magma intrusion and its propagation based on evolution of relocated seismicity^7^. The Bárðarbunga rifting event started on 16 August 2014 with magma migrating away from the caldera, first to the southeast and then to the northeast, accompanied with intensive seismicity^7^. The magma and the earthquake swarm propagated ∼40 km away from the caldera and passed the northern edge of the ice cap on 23 August (Fig. 1a). During the following 3 days, the seismicity progressed northward in a few pulses that lasted for several hours (Fig. 1b), separated by time periods of much lower seismic activity. In total, the activity propagated ∼13 km north from the edge of the glacier with a mean velocity of 400 m h^−1^ and a peak velocity of ∼1 km h^−1^. Most of the earthquakes and the seismic moment release of the entire dyke intrusion occurred at 6–9 km depth (Fig. 1c)^7^ and in <1 day during the main propagation pulse (grey rectangle, Fig. 1b,c), which likely coincided with the initiation of the graben subsidence at surface. Fault plane solutions of all *M*_w_>3.5 earthquakes consistently show predominant strike-slip mechanisms, with a partial normal faulting component (Fig. 1d; Supplementary Fig. 1). Although the dyke stopped its northward propagation on 26 August, the seismic activity continued, mostly at the eventual eruptive site and near the edge of the glacier.

**Figure 2:**
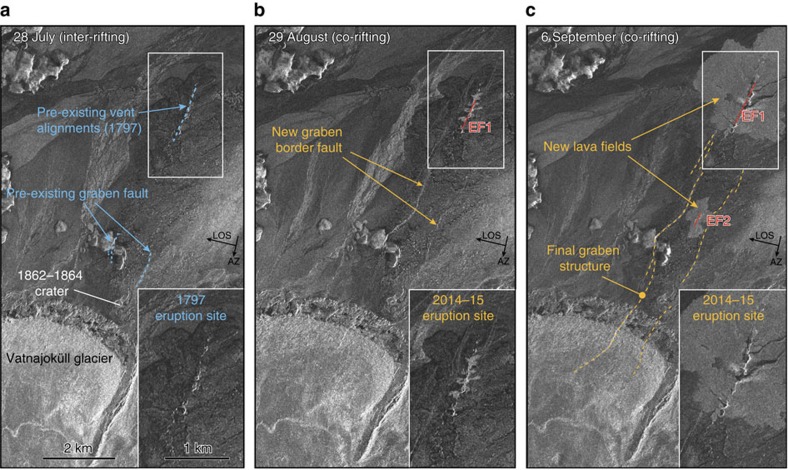
Volcano-structural evolution of the graben area. Three high-resolution satellite (COSMO-SkyMed) radar-amplitude images from (**a**) 28 July 2014 (pre-eruption) showing pre-existing graben faults and eruptive vents (in blue); (**b**) 29 August 2014 showing evidence of new graben faulting (this image was acquired at 19:37 GMT, ∼19 h after the first eruptive activity), and (**c**) 6 September showing the main eruptive vent (eruptive fissure 1; EF1) that was active for 6 months and the eruptive fissure 2 (EF2) that was active on 5 September 2014. White rectangle insets show the main eruption site in more detail.

The first sign of an eruption was observed on 29 August 2014 at 00:02 GMT (EF1, Figs 1b,d and 2b), about 13 days after the rifting event started. This brief and weak eruptive activity ceased after a few hours but then restarted on 31 August 2014 at 5:50 hours with much greater vigour with a mean discharge rate of ∼100 m^3^ s^−1^ over the 6-month eruption duration^9^. Another localized eruptive fissure opened to the south on 5 September 2014 in the morning for a few hours, producing a small intra-graben lava field (EF2, Figs 1d and 2c). The principal eruptive fissure built up a 500-m-long and ∼70-m-high spatter edifice that hosted a lava lake that fed the lava field, which reached a volume of ∼1.6 km^3^ by the time the eruption ended on 27 February 2015 (ref. 9).

### Graben deformation and modelling

We analysed the evolution of the graben and the deformation north of the glacier using multiple high-resolution radar images acquired from 28 July to 12 October 2014 (Supplementary Table 1). The volcanic vents from the 1797 eruption, as well as evidence for pre-existing graben faults on a topographic high can be seen in an image preceding the intrusion (Fig. 2a). Then, an image acquired on 29 August, about 19 h after the first eruptive activity began, shows that the 2014 eruption used precisely the same vents as the 1797 eruption (Fig. 2b). This image also shows the new graben faulting as bright linear features, fracturing the 1797 lava field, which indicates that the entire graben had been reactivated (Fig. 2b). This reactivation started before the small eruption on 29 August, as new graben faulting is seen in an air photo from 27 August^10^. The radar images show an overall narrow graben (mostly 500–800 m wide) bounded by two sub-parallel border faults that converge to the North towards the main eruption site (Fig. 2b). Subsequent images show an increase in the reflectivity of the border faults, most likely due to increasing fault throw, as well as both eruptive fissures and the new lava fields (Fig. 2c).

**Figure 3:**
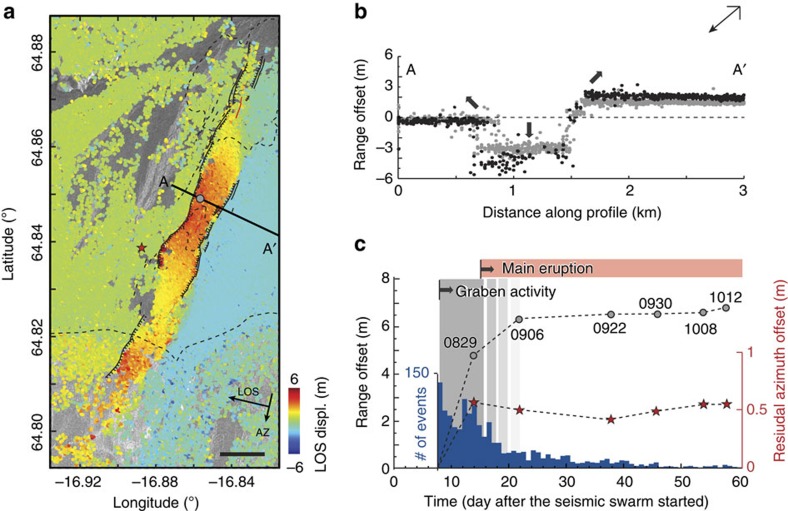
Graben deformation. (**a**) SAR-image (COSMO-SkyMed; descending orbit) range offsets of the graben area (29 August 2014) showing the line-of-sight (LOS; black arrow) ground displacements. Red indicates ground displacement down and away from the radar. Black lines mark the graben faults. Horizontal black scale bar, 1 km. (**b**) LOS displacement along profile A–A′ (location shown in **a**) for 29 August (grey dots) and 6 September (black dots) 2014. Thick arrows indicate subsidence and uplift directions; the down-looking arrow to the top right shows the satellite's LOS direction. (**c**) Displacement time series of the graben (location marked in **a** with a circle) for six different dates in comparison to the number of seismic events (histogram). The red stars are the azimuth offset time series after model removal (Fig. 4b), showing that the left-lateral shear occurred before 29 August. The progressively lighter grey shading with time illustrates the decaying graben deformation.

We produced six different near-field deformation maps by calculating pixel offsets between the radar images and then derived displacement time series along the directions of the satellite flight path (azimuth) and the radar line-of-sight (LOS; range)^11^ (see Methods and Supplementary Fig. 2). The data from the 29 August 2014 deformation map show an increase of up to 6 m in LOS displacement due to subsidence within the graben and a decrease of 3 m in LOS displacement (due to uplift and eastward displacement) east of the graben border fault (Fig. 3a). Little LOS displacement is seen west of the graben, as the westward motion is mostly counterbalanced by the graben flank uplift, in this descending-pass LOS imaging geometry. To further identify the graben structure, we extracted displacement profiles across the graben for two dates (29 August and 6 September 2014) and they show that the central graben subsidence increased by about 1 m between the two acquisitions (Fig. 3b). The overall deformation and graben subsidence are also clearly visible in a three-dimensional surface displacement field (Supplementary Fig. 3e), derived using radar-image offsets from both ascending and descending orbits, respectively, acquired on 4 and 6 September 2014. We then analysed the temporal evolution of the graben deformation by selecting an area within the graben that well-represents the general deformation trend. Our results indicate that most of the graben subsidence occurred during the first period (before 29 August) and then gradually slowed down into mid-September, together with a decay of the seismicity (Fig. 3c). No significant near-field deformation is observed after 22 September 2014, even though the eruption continued, suggesting that the active feeder dyke had reached its final thickness. This lack of near-field deformation also indicates that the eruption was entirely fed by the magmatic reservoir under Bárðarbunga caldera where intensive seismic activity and subsidence continued until the end of the eruption.

**Figure 4:**
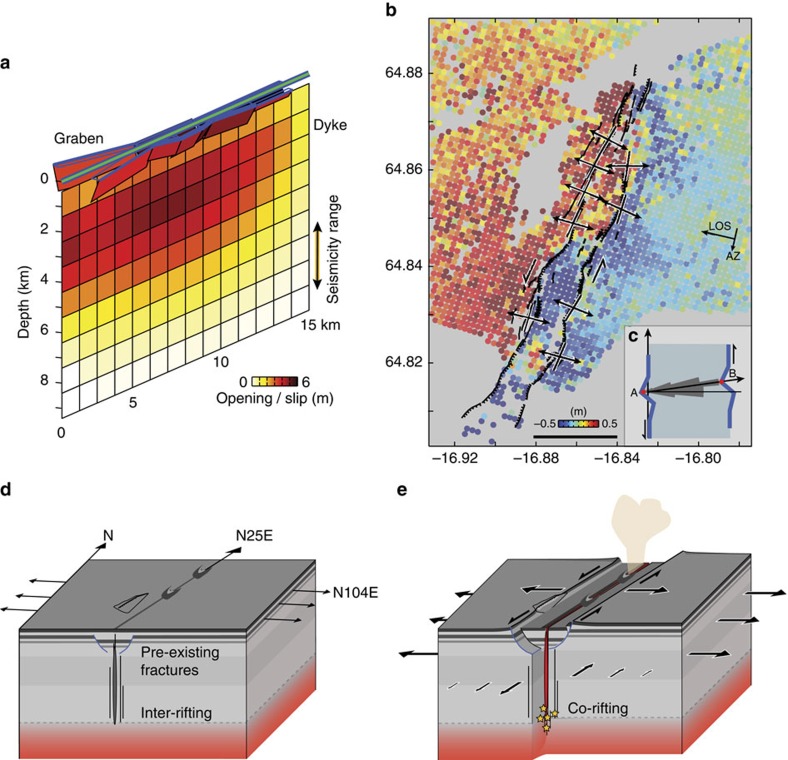
Dyke model and left-lateral shear opening. (**a**) Model of the dyke opening and graben faulting estimated from the radar-image offset data (Fig. 3a and Supplementary Fig. 3). Green line shows the surface projection of the dyke. (**b**) SAR-image residual azimuth offsets (6 September 2014) after removal of the dyke opening contribution (see modelling details in Supplementary Fig. 3), showing about 1 m of left-lateral shear across the graben. The black arrows show opening orientations across graben-bounding fractures measured in the field. Black horizontal bar scale at the inset bottom, 2 km. (**c**) Rose diagram (structural data set with *n*=108) showing the left-lateral shear opening; thick blue lines represent a schematic of an open fracture with matching asperities on the fracture walls (A and B showing the asperity match). (**d**) Schematic cross-section of the inter-rifting period subject to steady extension deficit accumulation oriented at N104° E. The pre-existing graben, which is buried below older lava flows, has an orientation of N25°E. The grey dashed line represents the brittle–ductile boundary. (**e**) Schematic cross-section of the 2014–2015 rifting event that shows the dyke intrusion causing near-field left-lateral shear, extension and graben reactivation. Yellow stars represent the earthquake locations at the lower edge of the dyke path.

The observed deformation can be modelled with a NNE-trending (25°) dyke parallel to the graben axis, with a maximum opening of 4.9 m at 3 km depth below the surface (the mean elevation at this location is ∼750 m) and a volume for this segment of ∼0.27 km^3^ (see Methods, Fig. 4a and Supplementary Fig. 3a–d). In our model, up to 5 m of slip on the normal faults is required to fit the graben deformation, which agrees with our field observations. Surprisingly, after removing the best-fit model prediction from the deformation data, an extensive left-lateral shear signal across the graben remains with an overall shear motion of about 1 m (Fig. 4b). This left-lateral shear across the graben was entirely released during the early phase of the dyke intrusion (before 29 August 2014) while the dyke opening kept increasing beyond 6 September 2014 (Fig. 3c).

To investigate this apparent left-lateral shear across the graben further, we visited the area in August 2015 and carried out field analysis of the graben-bounding surface fractures. We systematically measured the strike of 108 fractures and then their aperture orientations by matching asperities on the fracture walls (Supplementary Fig. 4). The mean opening direction is at an 83° angle to the strike of the fractures, but not perpendicular to them, indicating a consistent left-lateral oblique opening of the entire graben (Fig. 4c) in agreement with the satellite and seismic data.

## Discussion

The rifting activity in 1797 caused extension with normal faulting and graben subsidence, which later was mostly buried by the lava of the 1797 eruption (Figs 2 and 4d). That rifting event was most likely first followed by a post-rifting transient and then by an inter-rifting phase, during which extension deficit accumulated at a steady rate in the N104° E direction, that is, about 19° oblique to the graben (Fig. 4d). Another eruption probably occurred in 1862–64 within the southernmost section of the graben near the edge of the glacier^8^. However, this event does not seem to have reactivated the graben structure, suggesting that magma did not propagate as far north as in 1797. Then, during the subsequent rifting activity (from August 2014 to February 2015), the magma used the same intrusion pathway as in 1797, reactivating the same fractures and erupting at the same location. The intrusion therefore reactivated the graben and released the inter-rifting extension deficit accumulated during the past two centuries at this latitude, resulting in both opening and shear across the graben (Fig. 4e). By assuming a steady divergent plate motion rate of 18.5 mm per year at the graben location^1^ since the eruption 217 years ago, about 4 m of extension deficit accumulated during the inter-rifting period, which is in accordance with the 4.5 m widening observed at the surface during the 2014–15 rifting event. In addition, the estimated accumulated left-lateral shear deficit since the last eruption is ∼0.7 m, comparable to the 1 m of left-lateral shear we observed across the graben during the intrusion.

The rifting cycle described above (the 1797 dyke intrusion, relaxation, stress build-up and the new 2014–15 intrusion) is comparable to those proposed for Dabbahu and Krafla^4^,^5^ and such cycles are responsible for the morphology seen in rift zones. We propose that pre-existing fractures within and beneath graben structures play a dominant role in controlling the magma propagation and re-distribution of stresses during the rifting cycle. We find that the 2014–15 dyke, the graben and the eruptive fissures are parallel to one another, but all oriented obliquely to the regional stress field, which is commonly observed in rift zones^12^,^13^,^14^,^15^. This obliquity between the intrusive zone and the regional stress field may be due to the magma intruding into a pre-existing weak zone or it may be related to topographic loading, which has proven to influence dyke propagation^7^,^16^,^17^. Overall, and as recently suggested^14^, using fault zones and associated eruptive fissures as stress indicators should be done with caution. However, we have shown here that measuring fracture opening orientations is a powerful approach to retrieve information about the faulting kinematics. This method should be particularly useful for studying past rifting events for which no geodetic or seismic data are available.

Earthquakes associated with dyke propagation are usually linked to the evolution of dyke-induced stress changes and to the stress previously stored in the crust before the intrusion^18^,^19^,^20^. The seismicity associated with the Bárðarbunga intrusion is located well-below the shallow graben faulting we observed and modelled (Figs 1c and 4a), which indicates that both the graben faulting and the subsidence were aseismic. Most of the earthquakes occurred at 6–9 km depth^7^ or at a slightly shallower depth range of 5–7 km^21^, roughly corresponding to the lower edge of the modelled dyke (Fig. 4a). The absence of seismic activity above 5–6 km depth therefore supports our hypothesis that pre-existing fractures control the magma propagation through the shallow crust. This also indicates that oblique surface faulting can be used as a proxy to learn more about deeper magma-tectonic processes along rift zones.

The fault plane solutions have one of the nodal planes oriented sub-parallel to the intrusion and the graben (see Fig. 1c), indicating left-lateral shear faulting similar to the shear motion observed at surface in the SAR offset data and in the field. However, detailed moment tensor analysis of multiple earthquakes shows that the left-lateral failure planes are oriented ∼13° clockwise to the dyke strike, which may be explained by new dyke-tip fracturing in presence of high-fluid pressures^21^. The earthquakes thus may be due to new fracturing at the brittle–ductile boundary^22^ and at the lower edge of the pre-existing dyke path (Fig. 4e). The narrow depth range of the seismicity below the dyke could represent strain-rate hardening^22^ and brittle failure of the normally ductile material below the rapidly progressing magma intrusion. The observed seismicity therefore suggests that the magma opened pre-existing fractures and propagated aseismically at shallower depths and that those rapidly opening fractures extended into the brittle–ductile boundary causing new fracturing.

Based on the observations and results above, we thus posit that reactivation of pre-existing fractures is a key parameter for rifting mechanisms and dyke emplacements along divergent plate boundaries. We further suggest that the fracture reactivation initiates at the front of the propagating dyke and first releases both shear and opening, under the influence of the regional stress field, followed by further dyke opening. Finally, our findings directly challenge current dyke propagation models that classically do not consider pre-fractured medium.

## Methods

### Seismicity

The earthquake data shown in Fig. 1 are relocated earthquake locations reported by Sigmundsson *et al*.^7^ using data from the SIL (South Iceland Lowland) national monitoring network^23^. Focal mechanisms are best fitting solutions based on P-wave polarities, as well as P-, SH- and SV-wave amplitudes, using the approach of Slunga^24^. The selected mechanisms require a minimum of 5 fitting P-polarities and 10 stations with both P- and S-wave amplitudes.

### Synthetic aperture radar data processing

We processed 14 COSMO-SkyMed (CSK) images from the Italian Space Agency (ASI) and 2 TerraSAR-X (TSX) images from the German Aerospace Centre (DLR) to map the graben faults and the near-field deformation. The CSK images were acquired from descending orbits with pixel spacing of 2.1 m in azimuth and 0.9 m in range (the LOS direction) while the TSX images were acquired from ascending orbits with pixel spacing of 1.7 m in azimuth and 0.9 m in range (Supplementary Table 1). We co-registered the two data sets separately using their orbital information^11^ and a digital elevation model (DEM) with a resolution of 25 m. We then produced a time series of geo-referenced, radar-amplitude images for the graben mapping based on the same DEM, but oversampled to a grid size of 5 m.

To estimate the image offsets, we calculated cross-correlations between spatially distributed sub-images of the co-registered radar-amplitude images. The peak location in the obtained cross-correlation surface indicates the offsets between two sub-images in two dimensions^25^. The CSK and TSX range offsets measure ground displacement in their radar LOS directions, which are about 40° and 24° from the vertical with a component towards the west and the east, respectively, while the azimuth offsets measure horizontal along-track displacements, which are about SSW for the descending CSK and NNW for the ascending TSX images. We uniformly distributed cross-correlation windows with dimensions of 64-by-64 pixels to compute the image offsets due to the dyke intrusion and reduced the window size to 16-by-16 pixels in the near-field to improve the resolution of the derived displacements. Although using this smaller cross-correlation window results in more outliers in the offset estimation^26^,^27^, we were able to retrieve unprecedented deformation details along the two boundary faults and inside the graben.

### Deformation modelling

We first used a simple dyke model to generate synthetic surface displacements from which we created a quadtree sub-sampling mask to ensure a higher density of points near the graben^28^. We then sub-sampled the CSK (acquired on 6 September 2014) and TSX (acquired on 4 September 2014) offset measurements based on the quadtree mask, calculated variances of the offset values within each quadtree cell and used the variances as weights in the source model optimization.

We modelled the graben-bounding faulting and dyke intrusion with dislocation sources buried in a homogeneous and elastic half-space and estimated the amount of fault slip and dyke opening^29^. We fixed the geometry of the dyke to be vertical, NE-trending (25°) (like the seismicity) and passing through the central axis of the graben. The mapped surface fault traces in Fig. 1 were used as constraints for the location, length and strike of nine graben fault segments. We set the depth to the top of the dyke to ∼400 m and the dip of the graben-bounding faults to 60° after testing different options for these values. We then subdivided the dyke plane into 1 × 1 km patches and solved for variable opening of the dyke and slip on the graben fault planes using linear least-squares minimization with positivity and smoothness constraints.

### Data availability

The data that support the findings of this study are available from the corresponding authors upon request, except the seismic data. These seismic data from ref. 7 were obtained from the Icelandic Meteorological Office for the current study and are therefore not available from the authors directly.

## Additional information

**How to cite this article**: Ruch, J. *et al*. Oblique rift opening revealed by reoccurring magma injection in central Iceland. *Nat. Commun.* 7:12352 doi: 10.1038/ncomms12352 (2016).

## Supplementary Material

Supplementary Information Supplementary Figures 1-4 and Supplementary Table 1
