# Supplementary material for: Oblique rift opening revealed by reoccurring magma injection in central Iceland
Source: Nat Commun. 2016 Aug 5;7:12352. doi: 10.1038/ncomms12352 (PMC4980445; doi:10.1038/ncomms12352)
Supplement: Supplementary Information — Supplementary Figures 1-4 and Supplementary Table 1 [file ncomms12352-s1.pdf]

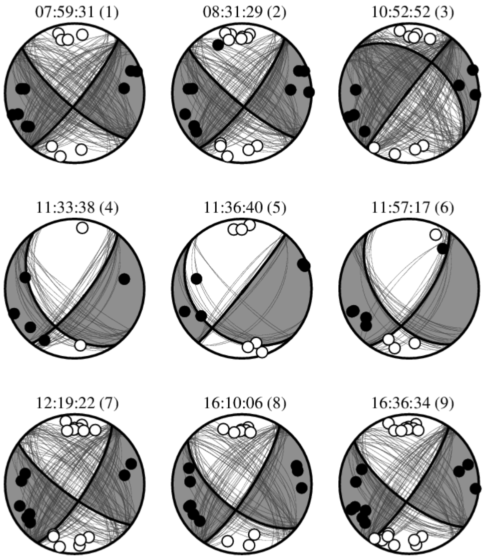

Supplementary Figure 1 | Lower hemisphere projections of focal mechanisms of nine selected earthquakes on 24 August 2014 as shown in Figure 1. The grey nodal planes give all possible mechanisms, the thick black nodal planes and the grey shaded beach ball in the background give the best fitting solution based on P, SV and SH amplitudes, as well as P-wave polarities. White dots give negative (downwards) and black dots positive (upwards) P-onsets movements on the vertical component. The average mechanism is strike-slip faulting. While the T-axes of the mechanisms are stably orientated in the E-W direction, the P-axes are not as well constrained, allowing for oblique normal components of some of the mechanisms. This slight instability is due to insufficient coverage of the area by the permanent SIL network. Ágústsdóttir et al. (2016) constrained almost pure strike-slip to be the dominant mechanism for similar events in the northern part of the dyke intrusion, using a dense local temporary network north of the Vatnajökull Icecap. Focal mechanisms are numbered as in the text / previous figures.

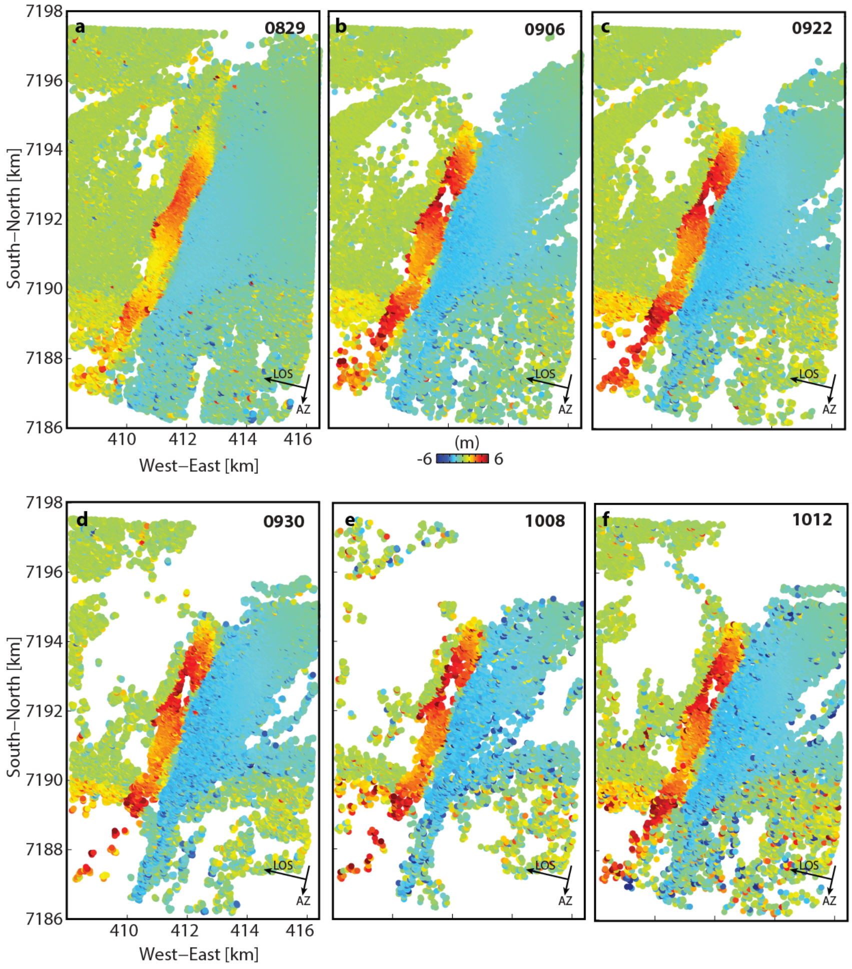

Supplementary Figure 2 | Graben deformation from 29 August to 12 October 2014. Range offsets of SAR-images (COSMO-SkyMed; descending orbit; master image is 28 July) of the graben area showing the ground displacement in the satellite line-of-sight (LOS; black arrow) direction for **a**, 29 August, **b**, 6 September, **c**, 22 September, **d**, 30 September, **e**, 8 October and **f**, 12 October 2014. Red colours indicate ground displacement down away from the radar. These data were used to produce the displacement time series shown in Fig. 3c, main text.

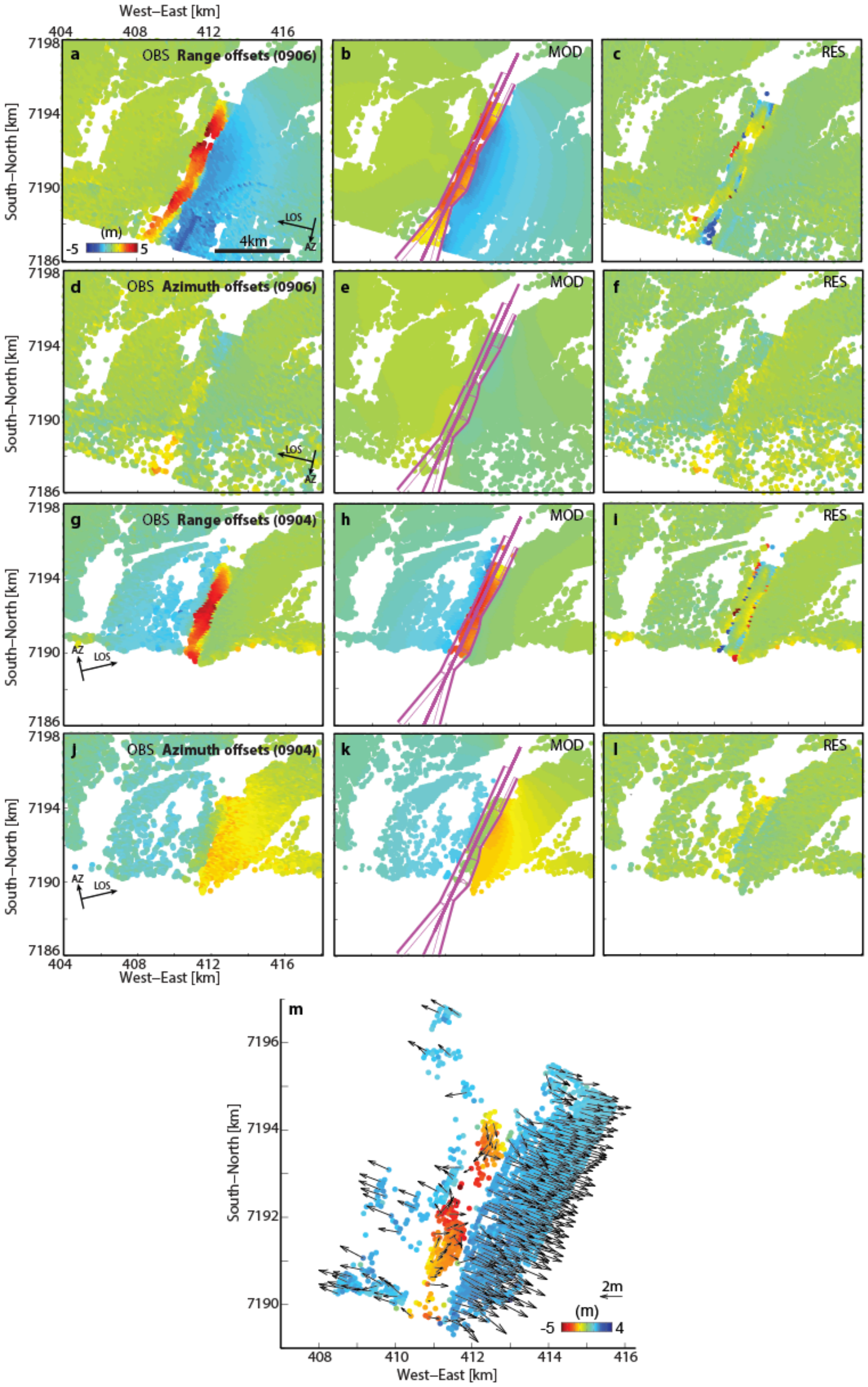

Supplementary Figure 3 | Dyke modeling and three-dimensional surface displacements. From a to f are the data (a, d), model predictions (b, e) and residuals

(c, f) for the 4 September 2014 descending COSMO-SkyMed range and azimuth offset data. From g to l are the data (g, j), model predictions (h, k) and residuals (i, l) for the 6 September 2014 ascending TerraSAR-X range and azimuth offset data. Red colours indicate ground displacement down away from the radar. **m**, Three-dimensional surface displacements derived using the range and azimuth offset data (a, d, g, j), with the arrows and the colormap showing the horizontal and vertical displacements, respectively; red indicates subsidence.

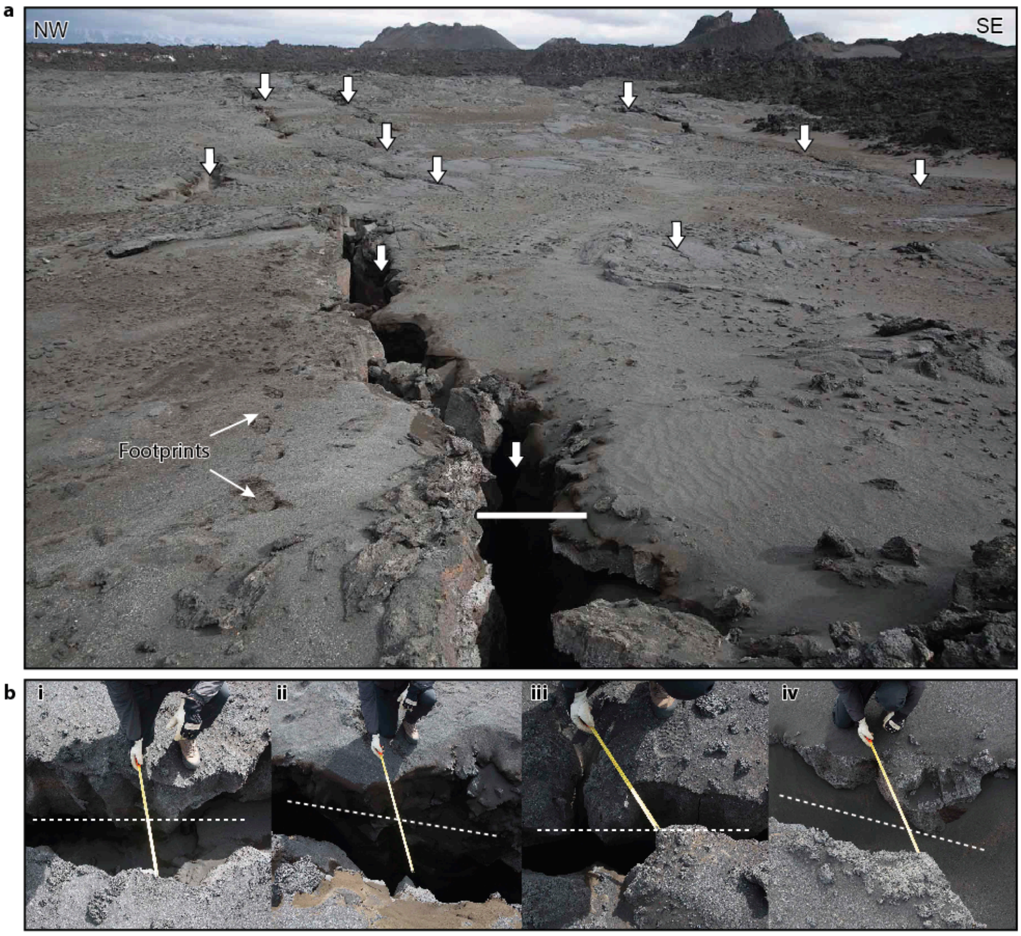

Supplementary Figure 4 | Structural field measurements along the graben. a, Typical fracture network with fractures highlighted by white arrows. See footprints on the left for scale; white horizontal bar is 50 cm at this location. **b**, subpanels i to iv are four examples of shear opening measured across fractures. The orientation of the fracture opening was measured with a compass by fitting asperities on the fracture walls. The results show that the opening orientations (i.e. the yellow meter) include an oblique component (left-lateral sense of shear) with respect to the mean fracture orientation (white dashed line).

Supplementary Table 1 | High-resolution satellite radar images used in this study.

Satellite	Flight direction	Imaging mode	Orbit track	Acq. Date (yyyymmdd)
TerraSAR-X	Ascending	Stripmap	O147	20120726<br>20140904
COSMO-SkyMed	Descending	Stripmap	2631	20140728<br>20140813<br>20140829<br>20140906<br>20140922<br>20140930<br>20141008<br>20141012<br>20141024<br>20141025<br>20141028<br>20141101
